# Insights in the determination of saxitoxin with fluorogenic crown ethers in water

**DOI:** 10.1007/s00706-017-2074-x

**Published:** 2018-01-11

**Authors:** Bernhard J. Müller, Günter Mistlberger, Ingo Klimant

**Affiliations:** Institute of Analytical Chemistry and Food Chemistry, Graz University of Technology, Graz, Austria

**Keywords:** Crown compounds, Sensors, Fluorescence, Photoinduced electron transfer, Biotoxin

## Abstract

**Abstract:**

In this contribution, we present new insights and a critical discussion in the optical detection of saxitoxin using fluorophores with crown ethers. Fluorescence enhancement is caused by the reduction of photoinduced electron transfer upon complexation with the analyte. Our attempts to improve this detection method neither did yield a functioning sensor nor were the attempts to reproduce published data in this area successful. Due to the fact that only low concentrations of saxitoxin are available, multiple surrogates were investigated at high concentrations. However, no turn on response was observed. Moreover, a fluorescent decomposition product of saxitoxin that forms under UV light was discovered which was in our opinion misinterpreted as a sensor response by previous publications.

**Graphical abstract:**

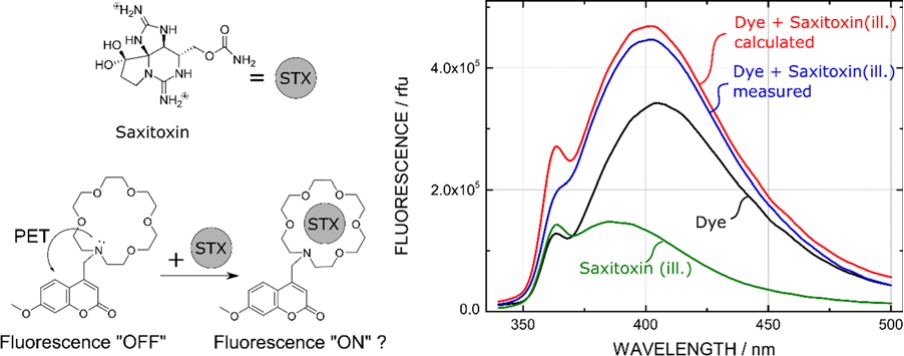

**Electronic supplementary material:**

The online version of this article (10.1007/s00706-017-2074-x) contains supplementary material, which is available to authorized users.

## Introduction

Saxitoxin (**1**) is one of the most toxic non-protein compounds known and is responsible for the so-called paralytic shellfish poisoning [1]. It is naturally produced by a variety of algal species, such as cyanobacteria and dinoflagellates, which are consumed in large amounts by shellfish during red tide algal blooms [2]. This accumulation of saxitoxin in shellfish which leads to the paralytic shellfish poisoning syndrome is a worldwide health problem. Saxitoxin acts as a sodium channel blocker by binding to a receptor site on the outer surface of the cell-membrane and inhibits the permeation of Na^+^ ions through the channel [3]. Consequently, action potentials are terminated and signal transmission between neutrons is inhibited, leading to paralysis [4].

Monitoring of this toxin by a mouse bioassay is used in many countries [5]. For ethical reasons, alternatives, such as HPLC methods were developed and are now routinely used [6–8]. The initial challenge in toxin detection is the lack of any UV absorption by saxitoxin. This can be overcome by oxidation of the toxin to a fluorescent derivate prior to or after separation on a HPLC column. The fluorescence can be observed at an excitation maximum of 330 nm and an emission maximum of 390 nm.

In recent years, a fluoroionophore-based method for the detection of saxitoxin was developed by the group of Gawley et al. [9]. This method was based on a commonly used concept for the measurement of cations, where a fluorescence indicator dye is linked to a recognition unit (e.g. crown ethers) [10]. Complexation of cations leads to a fluorescence enhancement caused by a reduced photoinduced electron transfer (PET) effect. It was assumed that saxitoxin could inhibit the PET effect as saxitoxin is a bis-guanidinium ion, and guanidinium ions are known to bind to crown ethers [11, 12]. Different crown receptors and indicator dyes were tested for their response to saxitoxin in this group with different fluorophores, e.g. anthracene [9, 12], coumarin [13–15], acridine [16], and aza-BODIPY [17]. We attempted to improve this method by preparing new indicator dyes and immobilizing these dyes into a polymer matrix, to obtain robust sensor films, enabling continuous measurements in aquatic media without pH interference. However, during our work we faced challenges in developing new optical sensors for saxitoxin, as well as reproducing published results [13]. Furthermore, we investigated the response of our sensors at high concentrations of different surrogates for saxitoxin and discovered an artefact, which could have compromised previous experiments and has to be avoided in the future. This will be discussed in this contribution.

## Results and discussion

The setup for a fluorescence optical sensor for saxitoxin is analogous to commonly used ion sensors. A fluorophore is linked to a recognition unit (receptor/ionophore) resulting in a fluoroionophore [18]. Typically, the receptor unit bears a tertiary amine group which is responsible for the emission enhancement in the presence of ions due to the reduced PET effect.

To date, receptors for saxitoxin detection were based on aliphatic aza-crown ethers. Those receptors are highly pH sensitive at physiological conditions because the amine can be easily protonated, which would result in a fluorescence enhancement similar to analyte binding. Moreover, most of the fluorophores which were used for the optical detection of saxitoxin were excitable in the UV region (330–390 nm), which can cause fluorescence background from biological samples (e.g. shellfish extract). Additionally, for measurements in the required low concentration ranges, the complex stability of crown ethers with analytes may be too weak in aqueous solutions. Complex stabilities in organic solvents are typically better and may be sufficient; however, usually aqueous conditions are required for the measurement of environmental samples.

To improve the commonly used setup, we introduced a lariat ether at the *ortho* position with respect to the nitrogen atom of the crown. This increases the binding efficiency of the analyte, since the two additional oxygen atoms also participate in the complexation [19]. We also decided to use an aromatic crown ether (substituted aniline), which is not sensitive to pH in the relevant range, since the p*K*_A_ value of the tertiary amine is ~ 5.5. As indicator, we used a commonly known BODIPY fluorophore which is excitable at > 400 nm, possesses a high photostability and molar absorption coefficient, and shows a high quantum yield.

Using this new indicator, the response to saxitoxin in solution was tested under similar conditions as in previously published work (H_2_O/EtOH/THF mixture, phosphate buffer at pH 7.2). A high fluorescence enhancement is obtained upon protonation of the amine group of the aza-crown ether indicating that the PET effect is suppressed. However, treating with saxitoxin did not show any fluorescence enhancement (Fig. S1, ESI). This negative result raises two fundamental questions: (1) is the complex stability (*K*_D_) of the complexation of saxitoxin in the crown ether sufficient to detect saxitoxin in the micromolar range? (2) If saxitoxin is complexed, does it suppress the PET effect or have any other influence on the photophysical properties?

**Fig. 1:**
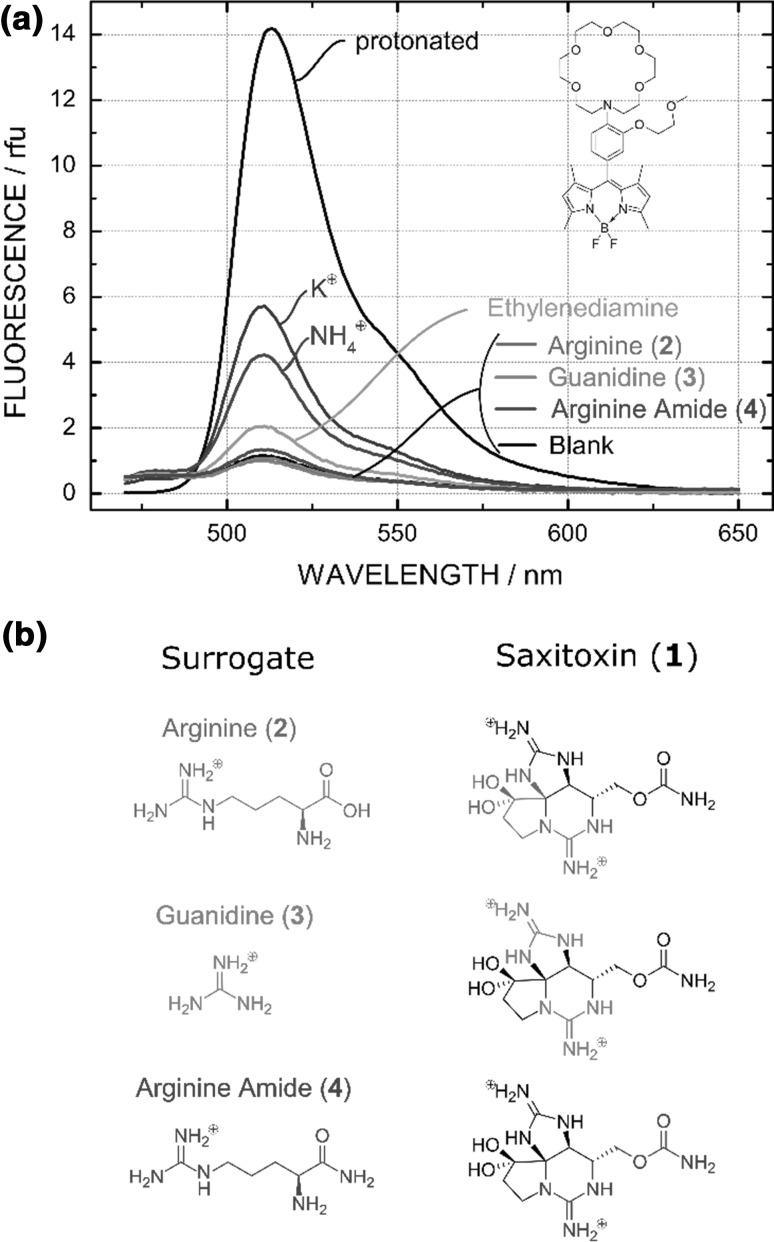
**a** Normalized emission spectra of the BODIPY fluorophore (10^−8^ M) with surrogates (10 mM) in a mixture of H_2_O/EtOH/THF (2/1/1) at pH 7.2. **b** Structurally similar surrogates **2**–**4** used to simulate saxitoxin (**1**) at higher concentrations (10 mM)

Since the concentration of saxitoxin is limited by the certified reference material (6.63 × 10^−5^ M stock solution) and it is not possible to obtain saxitoxin in higher concentrations, we investigated if a fluorescence enhancement can be obtained using structurally similar compounds at higher concentration (200× higher). Figure 1b summarizes the surrogate compounds **2**–**4** used to simulate saxitoxin as they are all subunits of saxitoxin itself and cover the whole molecule. Above all, guanidinium (**3**) is known to have a high binding affinity to 27-crown-9 and was proposed in previous work to be the structural compound of saxitoxin to inhibit PET [11, 17]. Additionally, we evaluated K^+^ and NH_4_^+^, and ethylenediamine because they are known for their binding affinity to the 18-aza-6-crown.

A high fluorescence increase can be observed in the presence of 10 mM K^+^ or NH_4_^+^, whereas a less pronounced response is caused by ethylenediamine (Fig. 1a, Fig. S2 ESI). However, we did not obtain any significant increase in fluorescence upon adding the surrogates. The same experiment was conducted in a DMSO/H_2_O (4 + 1) mixture as it is known, that the PET effect is more pronounced in more polar solvents. Again no significant increase of fluorescence using these surrogates was observable (Fig. S3, ESI).

The complexation behaviour is highly depending on two factors—the solvent and the size of the cavity and the analyte. Generally, crown ethers show the highest binding constants in methanol and the lowest in aqueous solution as a higher ratio of organic solvents are beneficial for the complexation [20]. The low complexation in water is due to a too-strong tendency to undergo hydration of the ion instead of getting complexed as the hydration shell around the ion needs to be stripped off [21]. Methanol or other organic solvents are much weaker solvating mediums and therefore hydration competes less with complexation yielding stability constants around three to four decades more than in water.

Another important parameter besides the solvent is the size of the crown cavity and the guest ion. As size of the 18-crown-6 is between 2.6 and 3.2 Å, it shows optimal interaction with K^+^ ion (2.66 Å) and NH_4_^+^ (2.86 Å) [21]. The corresponding stability constants of these in H_2_O are lg*K* = 2.05 for K^+^ and lg*K* = 1.44 for NH_4_^+^ [20, 22]. As the ammonium ion is substituted higher, the stability constants lower since the ion gets sterically hindered to fit into the crown ether [22]. This trend is observable in our data for K^+^, NH_4_^+^, ethylenediamine, and the surrogates. The amine group of the latter is highly substituted which consequently prevents the complexation.

However, reported saxitoxin-sensitive fluoroionophores which were used in aqueous solution show a binding constant 1000 × higher than for K^+^ [13, 14, 16]. Additionally, it was reported that complex stabilities of saxitoxin are higher in pure H_2_O than in an EtOH/H_2_O mixture, which is in contrast to the trend of measured binding constants of all crown–ion interactions in different solvents [13].

The published utilized fluoroionophore for saxitoxin measurements in water is based on a methoxycoumaryl-aza-crown dye, with which it was possible to measure the concentrations of saxitoxin in the micromolar range with 137 mM NaCl and 2.7 mM KCl as background [13]. Na^+^ and K^+^ did not “turn on” the sensor even though an aza-18-crown-6 was used as the recognition unit. In this work, saxitoxin binds to the receptor and inhibits the PET in a K^+^ background that is 27 times higher, whereas K^+^ does not turn on the sensor.

**Fig. 2:**
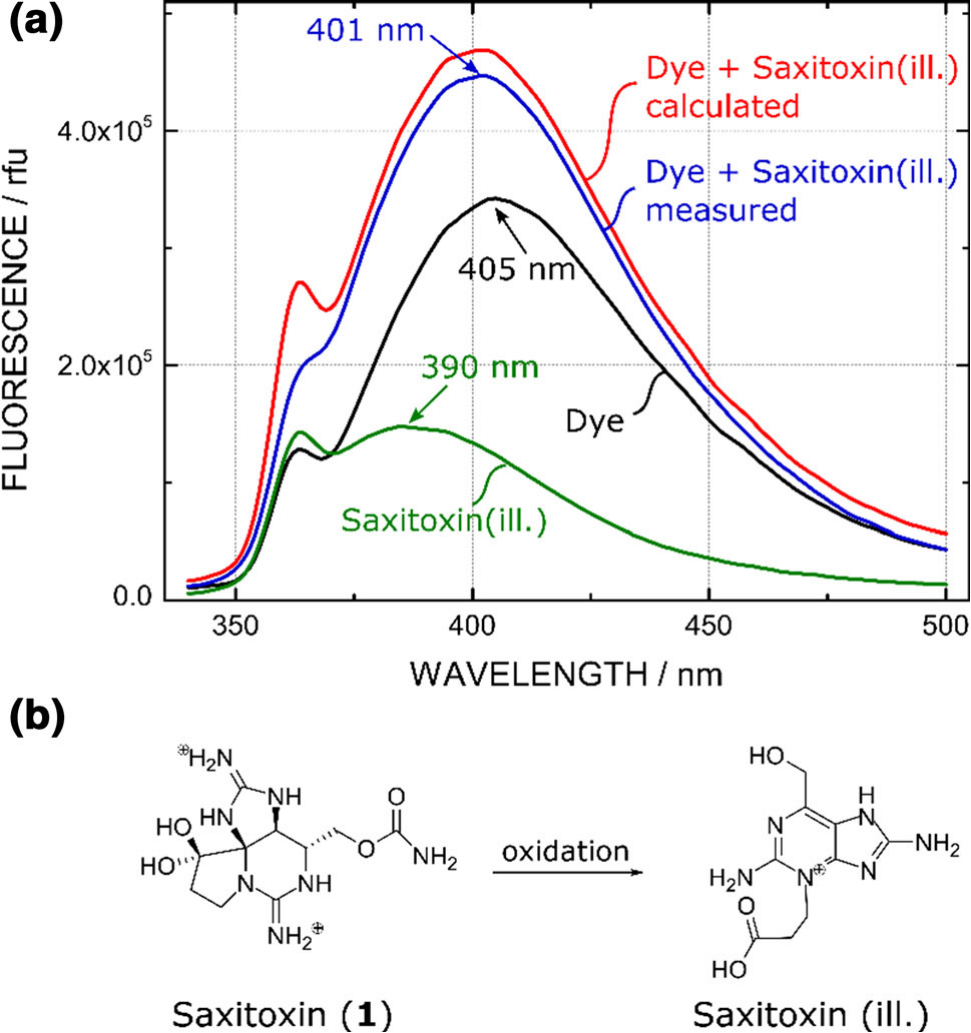
**a** Fluorescence spectra of saxitoxin (**1**) (1.6 **×** 10^−5^ M), the coumarin dye (10^−6^ M), the dye + saxitoxin measured, and the dye + saxitoxin calculated. The measured fluorescence enhancement of dye + saxitoxin is not based on the complexation of saxitoxin but on the additional fluorescence background of the saxitoxin oxidation product. **b** Structure of saxitoxin and the fluorescence oxidation product

We synthesized this saxitoxin-sensitive coumarin indicator dye as described in literature, and response to saxitoxin was tested under conditions similar to those reported (Fig. 2a) [13]. However, we could not observe any immediate increase in fluorescence with saxitoxin, but observed an increase in fluorescence intensity and a slight blue shift over the course of 20 min, similar to the published work. However, as a blank sample without any indicator was measured, we detected that saxitoxin itself starts to fluorescence upon illumination at 330 nm. This emission at 390 nm is shifted compared to the coumarin emission at 401 nm and superimposition with the coumarin fluorescence could explain the blue shift of the emission of the probe which is untypical for PET-indicators (Fig. 2a). Excitation spectra and emission spectra of both the coumarin dye and the saxitoxin illumination product are very similar and overlap over a broad range (Fig. S4, ESI). The saxitoxin decomposition product shows excitation and emission peaks of 334 and 390 nm, respectively. This corresponds to the fluorescent decomposition product that is usually obtained during the pre- or post-column oxidation of the HPLC–fluorescence detection method, where saxitoxin is chemically oxidized to the fluorescent purine derivate **5** (Fig. 2b) [6, 7, 23]. From this experiment, we concluded that the increase of fluorescence is not caused by inhibition of the PET effect by saxitoxin. Instead, we were able to determine that this increase in fluorescence can be attributed to a photooxidation product of saxitoxin itself.

To investigate the formation of this fluorescent saxitoxin product, we recorded the emission spectra of a buffered solution (pH 7.2) of saxitoxin alone (Fig. S5a, ESI). When saxitoxin is stored without exposing to light, no increase in fluorescence is observed. In contrast, strong fluorescence is detectable after illumination with UV light. The fluorescence intensity of the oxidation product after the illumination of saxitoxin with different intensities in the fluorimeter clearly shows that the formation of this fluorescent saxitoxin product is highly dependent on the intensity of the applied UV light and that saxitoxin does not form this product by simple exposure to ambient air (Fig. S5b, ESI).

It should be stated that Gawley et al. also detected this background fluorescence of saxitoxin, but interpreted it as a trace impurity of the saxitoxin solution and did not observe its increase during their measurements [16]. In our opinion, attributing the fluorescence increase of saxitoxin using the coumarin indicator to PET inhibition is a misinterpretation of data. The saxitoxin product shows very similar excitation and emission spectra to the used coumarin indicator and an addition of both fluorescence spectra explains the observed fluorescence enhancement by saxitoxin. Moreover, fluorescence enhancement due to saxitoxin oxidation can explain the observed blue shift of the emission in our measurement and in literature which is untypical for PET-indicators [14, 15]. With this in mind and the comparison of published stability constants of saxitoxin–crown interaction with well-known ion–crown complexations, we believe that the measurement of saxitoxin using this method in water is not achievable in the environmental necessary concentration range.

However, crown ether sensors for saxitoxin based on other fluorophores have been developed and work at different excitation/emission wavelengths. For these probes, the fluorescence increase is not influenced by this background fluorescence [17]. It is also important to note, that Gawley et al. in their earlier contributions were using non-aqueous solutions or a very high percentage of organic solvents which would be beneficial for the complexation of saxitoxin and much higher saxitoxin concentrations were used for the measurements [9].

## Conclusion

A critical examination of the optical detection of saxitoxin using complex fluorophore indicators in aqueous media is presented. An attempt to improve the detection method using a new fluoroionophore which shows significant inhibition of the PET effect with K^**+**^ or NH_4_^**+**^ ions was not successful. Testing structurally similar compounds as saxitoxin in higher concentration also did not yield a positive result. When reproducing literature where saxitoxin was detected in aqueous solution using a coumarin indicator dye, we discovered an artefact which was misinterpreted as response to saxitoxin. This artefact can be attributed to the intrinsic fluorescence of a known oxidative degradation product that is usually observed after chemical oxidation. We were able to identify UV light to be the reason for this oxidation. With this finding, it is possible to directly detect saxitoxin using this catalytic photooxidation which is currently under investigation in our laboratory and will be presented in the near future.

## Materials and methods

Saxitoxin dihydrochloride (**1**) (6.63 × 10^−5^ M in 3 × 10^−3^ M HCl) was purchased as certified reference material from the National Research Council Canada (www.nrc-cnrc.gc.ca). KCl and NH_4_Cl were obtained from Roth (www.carlroth.com). l-Arginine monohydrochloride (**2**), guanidine hydrochloride (**3**), l-argininamide dihydrochloride (**4**), and ethylendiamine were purchased from Sigma-Aldrich (www.sigmaaldrich.com). All other chemicals were obtained from TCI-Europe (www.tcichemicals.com). Synthesis of the BODIPY indicator was conducted as reported elsewhere [24]. Synthesis of the methoxycoumaryl-aza-crown fluorophore was conducted similarly to that described in literature [13]. Luminescence spectra were measured on a Fluorolog-3 luminescence spectrometer (Horiba). Fluorescence kinetic measurements were performed in a stirred and sealed micro quartz-cuvette from Hellma (www.hellma-analytics.com). All measurements were performed in buffered solution (phosphate buffer, 50 mM, pH 7.2). Measurements with surrogates were performed in EtOH/THF/H_2_O (1/1/2) and DMSO/H_2_O (4/1) with a dye concentration of 10^−8^ M, surrogate concentration of 1 × 10^−2^ M and a phosphate buffer (pH 7.2, 50 mM).

## Electronic supplementary material

Below is the link to the electronic supplementary material.

Supplementary material 1 (DOCX 2965 kb)
