# Supplementary material for: Insights in the determination of saxitoxin with fluorogenic crown ethers in water
Source: Monatsh Chem. 2018 Jan 11;149(3):493–7. doi: 10.1007/s00706-017-2074-x (PMC5859042; doi:10.1007/s00706-017-2074-x)
Supplement: Supplementary file 1 — Supplementary material 1 (DOCX 2965 kb) [file 706_2017_2074_MOESM1_ESM.docx]

**Supporting Information**

Insights in the determination of saxitoxin with fluorogenic crown ethers in water

**Bernhard J. Müller^1^ ● Günter Mistlberger^1^ ● Ingo Klimant^1^**

b.mueller@tugraz.at

^1^Institute of Analytical Chemistry and Food Chemistry, Graz University of Technology, Graz, Austria


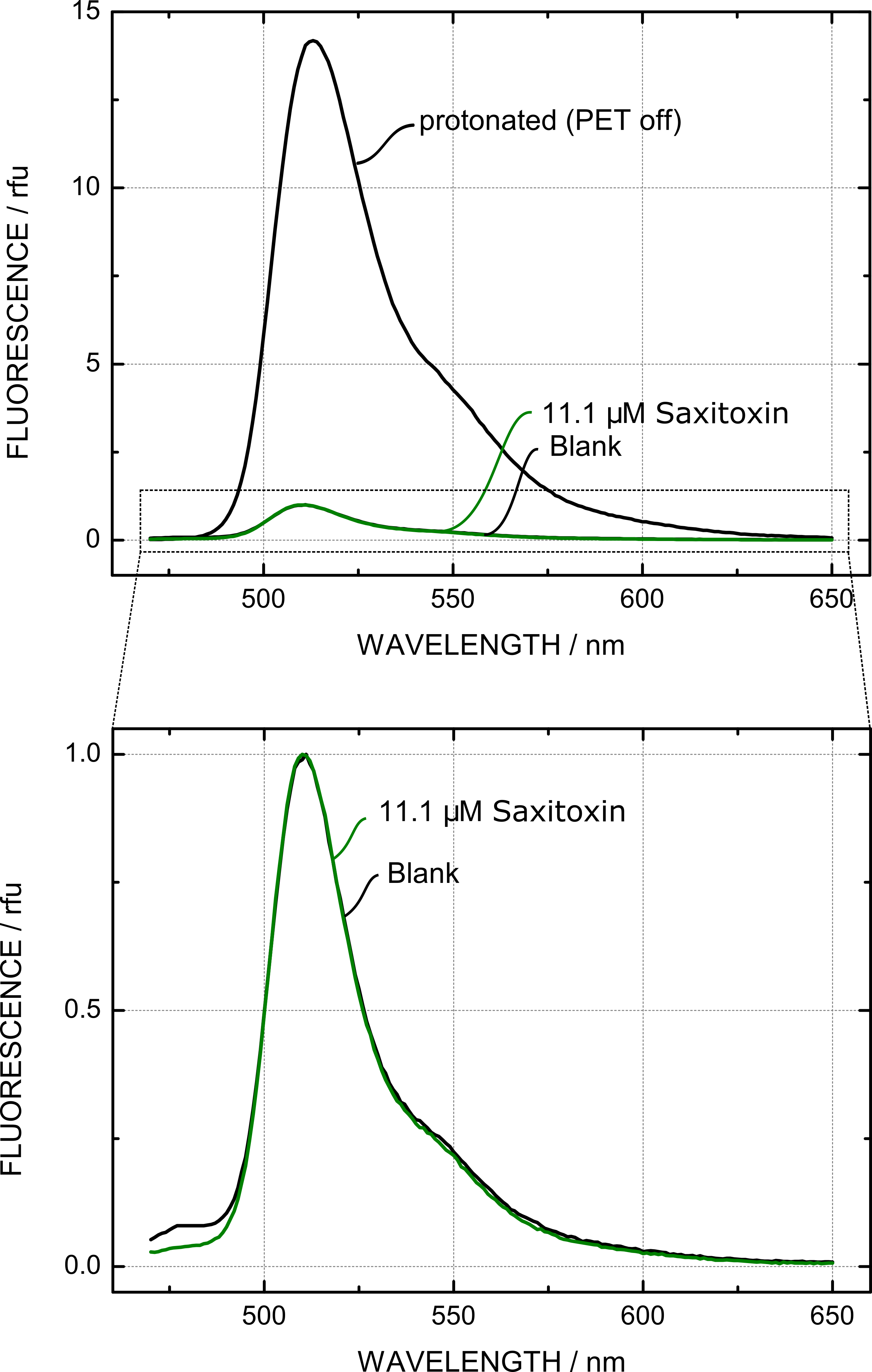


Fig. S1 Fluorescence response of the BODIPY fluorophore with saxitoxin. Top: Fluorescence can be turned on completely by acidification with HCl. Saxitoxin does not show any response in the micro molar range. Bottom: Close up to the emission spectra of the blank and saxitoxin.


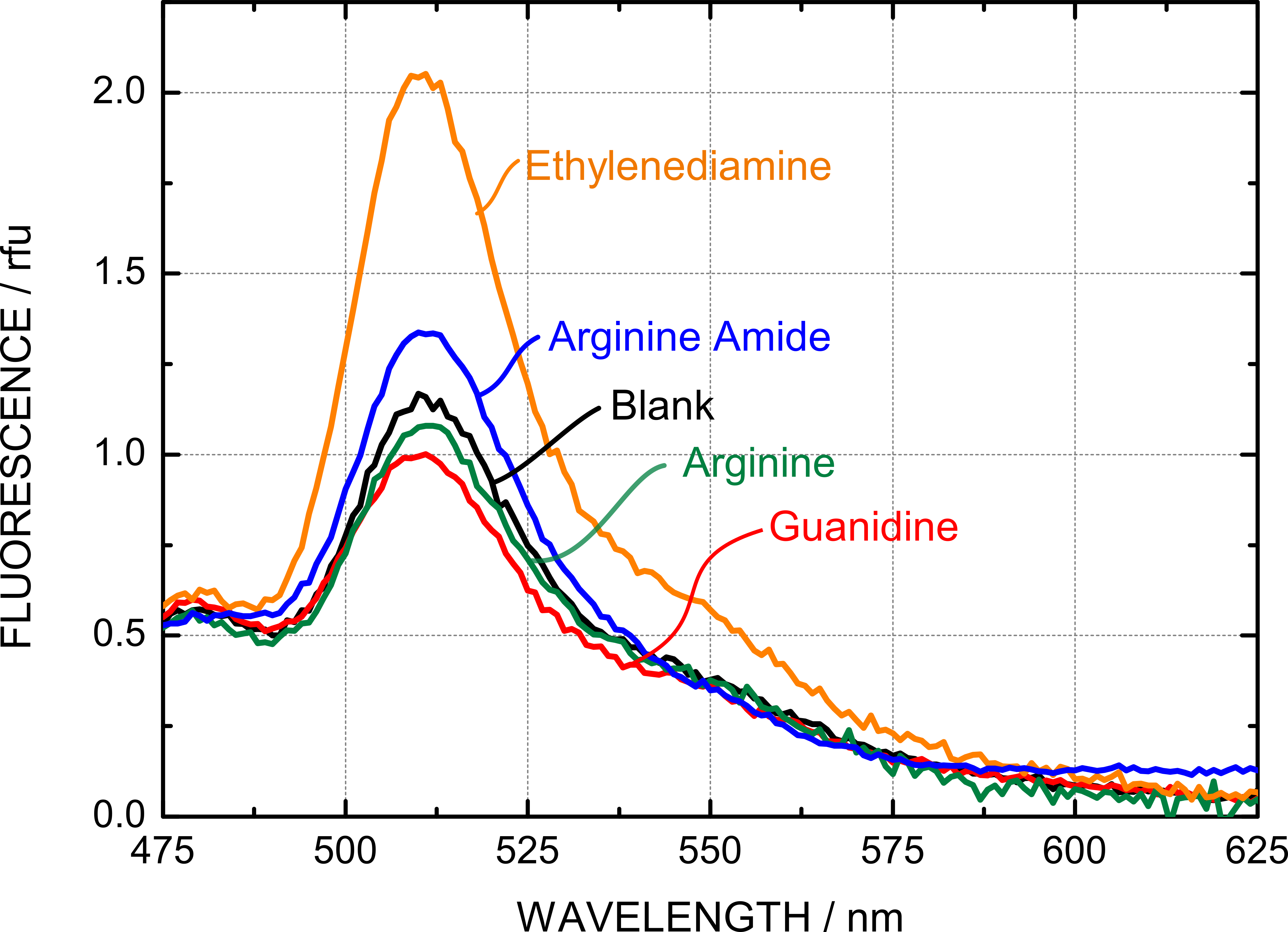


Fig. S2 Normalized emission spectra of the BODIPY fluorophore (10^-8^ *M*) with surrogates (10 mM) in a mixture of H_2_O/EtOH/THF (2/1/1) at pH 7.2.


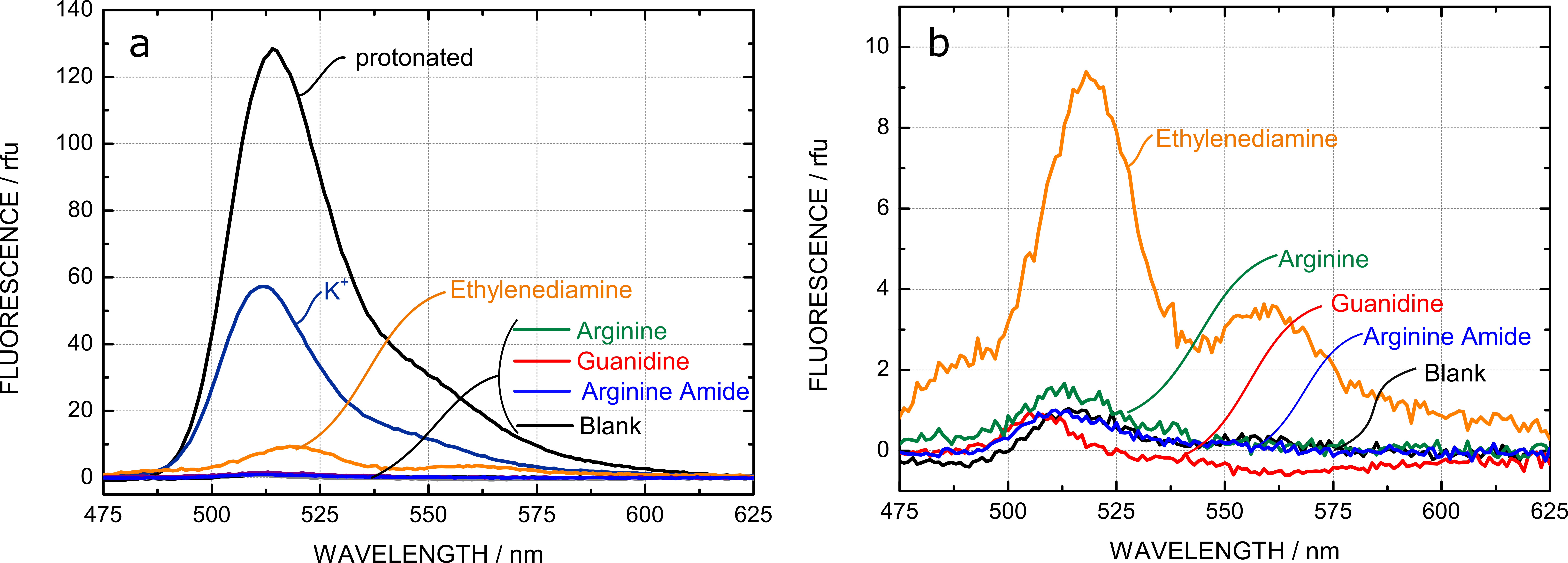


Fig. S3 (a) Normalized emission spectra of the BODIPY fluorophore (10^-8^ *M*) with surrogates (10 mM) in a mixture of DMSO/H_2_O (4/1) at pH 7.2. (b) Zoom in.


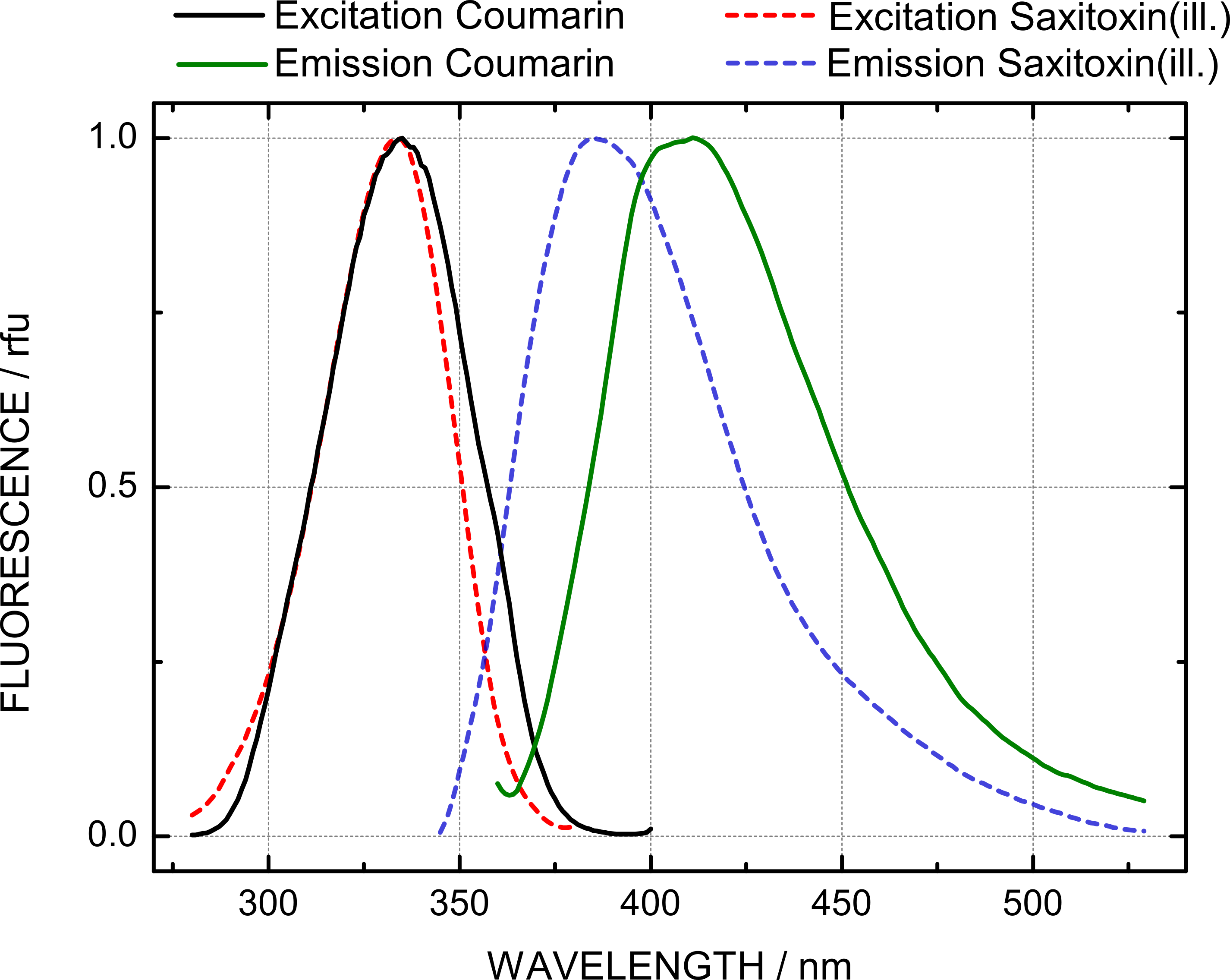


Fig. S4 Excitation and emission spectra of the coumarin fluorophore and saxitoxin degradation product after illumination.


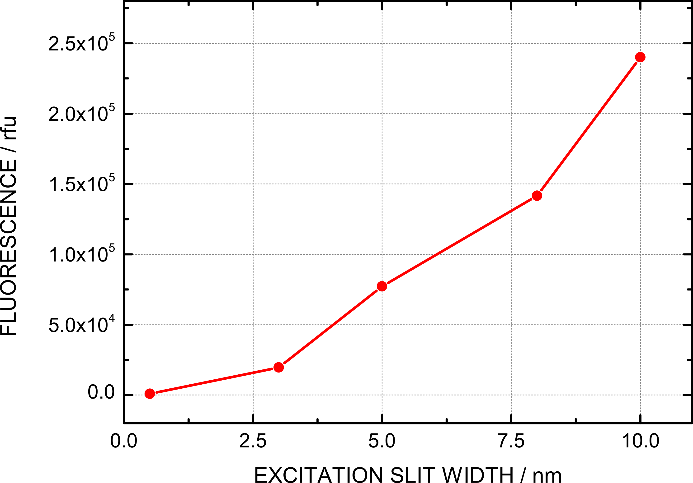

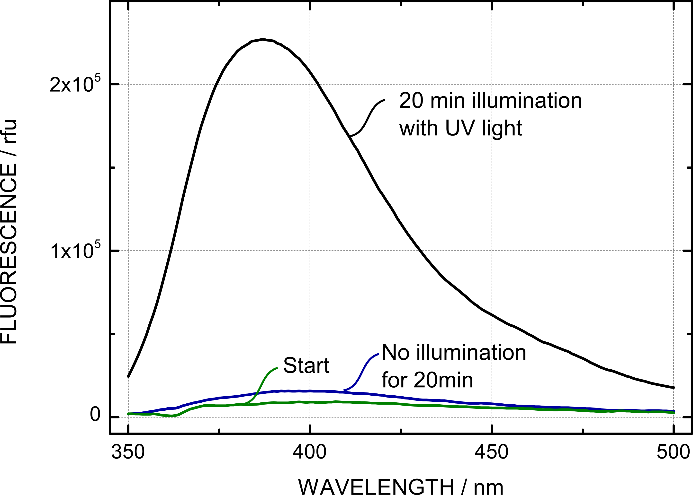


**A**

**B**

**Fig. S5 (a)** Emission spectra of a buffered solution of saxitoxin (1.6*10^-5^ *M*) after illumination and after storage in darkness. **(b)** Intensity of the emission maxima of the fluorescent product after excitation with different excitation slits (different UV light intensity).
